# Mycorrhizal-Mediated Lower Proline Accumulation in *Poncirus trifoliata* under Water Deficit Derives from the Integration of Inhibition of Proline Synthesis with Increase of Proline Degradation

**DOI:** 10.1371/journal.pone.0080568

**Published:** 2013-11-18

**Authors:** Ying-Ning Zou, Qiang-Sheng Wu, Yong-Ming Huang, Qiu-Dan Ni, Xin-Hua He

**Affiliations:** 1 College of Horticulture and Gardening, Yangtze University, Jingzhou, China; 2 NSW Department of Primary Industries, West Pennant Hills, New South Wales, Australia; 3 School of Plant Biology, University of Western Australia, Crawley, Western Australia, Australia; Key Laboratory of Horticultural Plant Biology (MOE), China

## Abstract

Proline accumulation was often correlated with drought tolerance of plants infected by arbuscular mycorrhizal fungi (AMF), whereas lower proline in some AM plants including citrus was also found under drought stress and the relevant mechanisms have not been fully elaborated. In this study proline accumulation and activity of key enzymes relative to proline biosynthesis (▵^1^-pyrroline-5-carboxylate synthetase, P5CS; ornithine-δ-aminotransferase, OAT) and degradation (proline dehydrogenase, ProDH) were determined in trifoliate orange (*Poncirus trifoliata*, a widely used citrus rootstock) inoculated with or without *Funneliformis mosseae* and under well-watered (WW) or water deficit (WD). AMF colonization significantly increased plant height, stem diameter, leaf number, root volume, biomass production of both leaves and roots and leaf relative water content, irrespectively of water status. Water deficit induced more tissue proline accumulation, in company with an increase of P5CS activity, but a decrease of OAT and ProDH activity, no matter whether under AM or no-AM. Compared with no-AM treatment, AM treatment resulted in lower proline concentration and content in leaf, root, and total plant under both WW and WD. The AMF colonization significantly decreased the activity of both P5CS and OAT in leaf, root, and total plant under WW and WD, except for an insignificant difference of root OAT under WD. The AMF inoculation also generally increased tissue ProDH activity under WW and WD. Plant proline content significantly positively correlated with plant P5CS activity, negatively with plant ProDH activity, but not with plant OAT activity. These results suggest that AM plants may suffer less from WD, thereby inducing lower proline accumulation, which derives from the integration of an inhibition of proline synthesis with an enhancement of proline degradation.

## Introduction

Water deficit (WD) or drought obviously limits plant growth and productivity, and plants possess a range of morphological, physiological and biochemical adaptive mechanisms in response to WD [1], [2]. Meanwhile, plants accumulate compatible osmolytes such as amino acids and their derivatives in response to WD, which protect plant cells from desiccation or dehydration but not interfere with biochemical processes [2], [3]. Proline, the most widely distributed osmolyte in higher plants, can be accumulated as a common metabolic response of osmotic adjustment to WD [4]. In plants, proline is synthesized mainly by the glutamate synthetic pathway in cytoplasm or chloroplast, which outlines that glutamate firstly converts into ▵^1^-pyrroline-5-carboxylate by ▵^1^-pyrroline-5-carboxylate synthetase (P5CS, EC 2.7.2.11/1.2.1.41, a key enzyme in the glutamate synthetic pathway of proline) and then transforms into proline by ▵^1^-pyrroline-5-carboxylate reductase (P5CR) [3], [4]. An alternative pathway, the ornithine synthetic pathway, shows that proline is synthesized from ornithine in mitochondrion, which is firstly transaminated by ornithine-δ-aminotransferase (OAT, EC 2.6.1.13, a key enzyme in the ornithine synthetic pathway of proline) to produce ▵^1^-pyrroline-5-carboxylate and glutamate-semialdehyde, and then converted to proline [4]. In mitochondria, proline is catabolised by proline dehydrogenase (ProDH, EC 1.5.99.8, a key enzyme in proline catabolism) into ▵^1^-pyrroline-5-carboxylate. As a result, the two proline synthetases, P5CS and OAT, and the proline catabolic enzyme, ProDH, are mainly involved in the net proline accumulation in plants.

Arbuscular mycorrhizal fungi (AMF) are able to form symbiotic associations with more than 80% of land plants for better uptake of nutrient and water [5]. Studies have shown greater drought tolerance in AMF-colonized citrus plants by a number of mechanisms. These mechanisms include enhanced water and nutrient uptake directly by extraradical hyphae, higher leaf stomatal conductance and/or better root system architecture [6], [7], higher capacity of osmotic adjustment and antioxidant defense systems [8]–[10], better soil structure due to higher glomalin [11], and over expression of genes encoding antioxidant enzymes [12].

In general, proline accumulation positively correlates with drought tolerance of AMF-colonized plants. Compared with the non-mycorrhizal plants, proline accumulation in the AMF-colonized plants exposed to WD was higher in *Lactuca sativa* [13], *Macadamia tetraphylla* [14], *Oryza sativa* [15], *Pistacia vera* [16], but lower in *Citrus tangerina* [9], *Erythrina variegata* [17], *Knautia arvensis* [18], and *Poncirus trifoliata* [19]. Moreover, mycorrhization in *Glycine max* induced higher proline accumulation in roots but lower in shoots under WD [20]. Augé and Moore [21] proposed that lower proline accumulation in AM plants was attributed to less injury by WD. It seems that proline accumulation may act as an appropriate indicator to evaluate the AM functioning on drought tolerance of host plants [22]. Further studies are thus needed to understand these discrepancies if a net proline accumulation relates to the activity of proline synthetases and/or catabolic enzymes in AM plants under WD.

Trifoliata orange (*Poncirus trifoliata* L. Raf.), a citrus relative, is relatively sensitive to soil water deficit and also is highly dependent on mycorrhizal symbiosis due to less root hairs. The objective of the present work was therefore to determine the relationships between proline accumulation and activity of key proline metabolic enzymes (P5CS, OAT and ProDH) in trifoliate orange under AMF colonization and/or soil water deficit.

## Materials and Methods

### Mycorrhizal inoculum

An AM fungus strain, *Funneliformis mosseae* (syn. *Glomus mosseae*) (Nicol. & Gerd.) Schüßler & Walker (BGC XZ02A) has been used since trifoliate orange has higher drought tolerance under the inoculation with this native strain than other *Glomus* species or strains [23]. This AM strain was originally isolated from the rhizosphere of *Incarvillea younghusbandii* in Dangxiong, China. The AM inoculum was propagated through the identified spores with white clover (*Trifolium repens*) for 16 weeks and was a mixture of AM-infected roots, spores (∼23 spores/g), hyphae and sand.

### Plant culture

Seeds of trifoliate orange were surface sterilized with 70% ethanol for 15 min and rinsed thoroughly with distilled water. On March 15, 2012, five sterilized seeds were sown in a 18 × 16.5×13 cm (top diameter × bottom diameter × height) plastic pot filled with 2.3 kg autoclaved (0.11Mpa, 121°C, 2 h) soils (Xanthi-udic Ferralsol, pH 6.1, FAO System). Soils at 0–20 cm depth were collected from a campus Citrus Orchard of Yangtze University, Jingzhou, China. This soil contained organic matter 9.8 g/kg, available N 114.2 mg/kg, Olsen-P 15.7 mg/kg, and available K 20.8 mg/kg. Before seed sowing, the AMF inoculation pots were received 40 g AM inocula at 5 cm soil depth, while the no-AMF pots were received both 40 g autoclaved (0.11Mpa, 121°C, 2 h) inocula and 2 ml inoculum filtrate (25 µm filter) for other microbial communities. After 40 days of sowing, three seedlings per pot were grown under 338–982 µmol/m^2^/s photosynthetic photon flux density, 25/19°C (day/night), and 70–95% relative air humidity in a plastic greenhouse locating in the university campus.

### Experimental design

The experiment consisted of a randomized block design with two AMF (with and without *F. mosseae*) and two water treatments (well-watered, WW; and water deficit, WD). The four treatments were coded as AM^−^/WW (no-AM and WW), AM^−^/WD (no-AM and WD), AM^+^/WW (AM and WW), and AM^+^/WD (AM and WD), respectively. Each treatment had three replicates, and each replicate (pot) contained three seedlings. A total of 36 seedlings (4 treatments × 9 seedlings/treatment) were used at harvest in the study. After 87 days of acclimation for mycorrhizal formation, the WW (100% of field water-holding capacity, corresponding to 23.9% soil moisture) and the WD (57% of field water-holding capacity according to Yang et al. [24], corresponding to 13.7% soil moisture) treatments were withheld for 80 days with pot weighing and then water supplement at an interval of two days from June 10, 2012 until harvest. During water treatments, the location of pots was weekly swapped to avoid possible environment effect.

### Variable analysis

Plant height, stem diameter, and leaf number per plant were measured before harvest. Shoots and roots of three seedlings from each replicate or pot were separately harvested as one composite sample and then dry weights were determined after 75°C for 48 h. A portion of fresh root segments were stained by 0.05% trypan blue [25], and root mycorrhizal colonization was expressed as the percentage of colonized root length with the observed total root length. The root systems were quickly scanned with an Epson Perfection V700 Photo Dual Lens System (J221A, Indonesia). Whereafter, the scanned images of root systems were analyzed using a WinRHIZO professional 2007b software (Regent Instruments Inc., Quebec, Canada) and root total length, surface area and volume were automatically obtained. Leaf relative water content (LRWC) was measured with the fourth fully expanded top leaf [26]. Analysis of leaf or root proline concentration (mg/g DW) was accorded to the acid-ninhydrine method [27]. The weighted plant total proline concentration was calculated as (leaf proline concentration × leaf biomass weight + root proline concentration × root biomass weight)/(leaf biomass weight + root biomass weight). Proline content (mg/plant DW) was the amount of corresponding tissue biomass × proline concentration.

Activity of leaf or root P5CS was determined according to the method of Hayzer and Leisinger [28] with minor modifications. Briefly, 0.25 g frozen tissues were homogenized with 6 ml 0.5 M Tris-HCl (pH 7.5) containing 10 mM MgCl_2_, 2 mM phenylmethylsulfonyl fluoride, and 2% (w/v) polyvinylpolypyrrolidone, and then centrifuged at 20,000 *g* for 20 min at 4°C. One ml supernatant was mixed with 3 ml 50 mM Tric-HCl containing 20 mM MgCl_2_, 10 mM ATP, 100 mM hydroxylamine-HCl and 50 mM glutamine. After 15 min at 37°C, the reaction was stopped by the addition of 3 ml 5 M HCl containing 5% FeCl_3_ and 12% trichloroacetic acid. The mixture was centrifuged at 20,000 *g* for 10 min at 4°C, and the absorbance of the supernatant was determined at 535 nm, with the no-ATP reactive solution as the control. One unit of P5CS was expressed as the enzyme amount of 1.0 μmol glutamate during 1 min (U/g DW). The weighted plant total activity of P5CS was calculated as (leaf activity of P5CS × leaf biomass weight + root activity of P5CS × root biomass weight)/(leaf biomass weight + root biomass weight).

Determination of leaf or root OAT activity was accorded to Kim et al. [29], and one unit of OAT was defined as the amount of 1 nmol ▵^1^-pyrroline-5-carboxylate during 1 min (U/g DW). The weighted plant total activity of OAT was calculated as (leaf activity of OAT × leaf biomass weight + root activity of OAT × root biomass weight)/(leaf biomass weight + root biomass weight).

Activity of leaf or root ProDH was assayed according to Zhao et al. [30] with minor modifications. Briefly, 0.25 g frozen tissues were homogenized with 6 ml phosphate buffer (pH 7.8) containing 1.0 M EDTA and 10 mM mercaptoethanol. After centrifugation at 4,000 *g* for 15 min at 4°C, 10 µl TritonX-100 was added, placed for 30 min at 4°C, and centrifuged at 20,000 *g* for 20 min at 4°C. A total of 2.5 ml solution consisted of 0.5 ml supernatant, 1.6 ml bicarbonate buffer (pH 10.3), 0.2 ml 0.1 mM proline, and 0.2 ml 0.9 mM 2, 6-dichlorophenolindophenol. After the addition of 0.2 ml 9 mg/ml phenazine methosulfate, the absorbance of the solution increased 0.01 at 600 nm during 1 min was defined as one activity unit of ProDH (U/g DW). The weighted plant total activity of ProDH was calculated as (leaf activity of ProDH × leaf biomass weight + root activity of ProDH × root biomass weight)/(leaf biomass weight + root biomass weight).

### Statistical analysis

Data (means ± SD, *n* = 3) were subjected to two-way ANOVA and Duncan’s multiple range tests were used to compare significant differences among treatments at the 5% level. All statistical analyses were performed with the SAS v8.1 software (SAS Institute Inc., Cary, NC, USA).

## Results

Root mycorrhizal colonization was significantly higher under WW (69.06±8.91%) than under WD (52.20±4.00%). No root mycorrhizal colonization was observed in the no-AMF inoculated seedlings, regardless of the soil water status.

The water deficit generally significantly decreased plant height, stem diameter, and leaf number, irrespective of AM or no-AM treatment (Table 1). The AM inoculation significantly increased plant growth performance under both WW and WD. The water deficit also significantly restricted root total length and surface area in AM seedlings but not in no-AM seedlings, and the AM seedlings recorded notably higher root total length and surface area under WW but not under WD. The water deficit did not significantly affect root volume regardless of AM or no-AM, whereas the AM inoculated treatment significantly increased root volume, irrespective of water status.

**Table 1:** Effects of *Funneliformis mosseae* on growth performance and root traits of 167-day-old *Poncirus trifoliata* under well-watered and water deficit (WD).

Treatments	Plant height (cm)	Stem diameter (cm)	Leaf number per plant	Root total length (cm)	Root surface area (cm^2^)	Root volume (cm^3^)
AM^−^/WW	27.3±1.3b	0.26±0.01b	23±1b	270±16b	42.4±3.6b	0.55±0.09bc
AM^−^/WD	23.2±1.6c	0.24±0.01c	18±1c	257±8b	40.1±4.0b	0.46±0.07c
AM^+^/WW	32.6±2.6a	0.29±0.01a	26±3a	440±10a	70.0±1.9a	0.84±0.11a
AM^+^/WD	26.8±1.1b	0.26±0.01b	21±1b	289±36b	46.6±16.8b	0.75±0.17ab
Significance						
AMF	**	**	**	**	*	**
WD	**	**	**	**	*	NS
AMF×WD	NS	NS	NS	**	NS	NS

Data (means ± SD, *n* = 3) followed by different letters among treatments indicate significant differences at 5% level. Abbreviations: AM^−^/WW, non-mycorrhizal and well-watered control; AM^+^/WW, mycorrhizal and well-watered; AM^−^/WD, non-mycorrhizal and water deficit; and AM^+^/WD, mycorrhizal and water deficit. *, *P*<0.05; **, *P*<0.01. NS: not significant.

**Figure 1:**
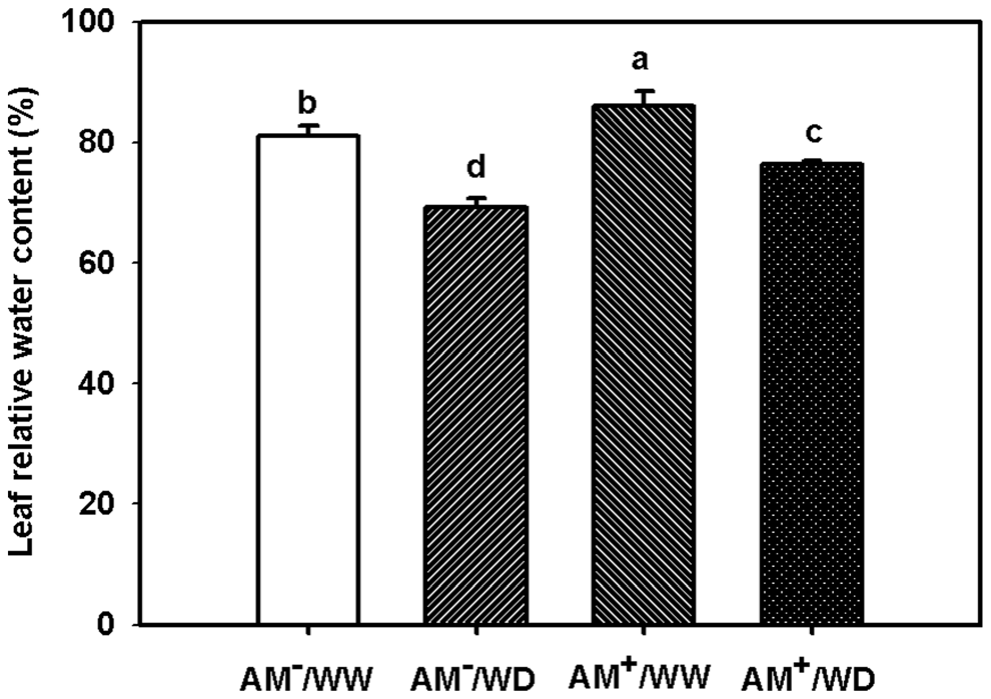
Effect of *Funneliformis mosseae* on leaf relative water content of 167-day-old *Poncirus trifoliata* under well-watered and water deficit. Data (means ± SD, *n* = 3) followed by different letters above the bars among treatments indicate significant differences at 5% level. Abbreviations: AM^−^/WW, non-mycorrhizal and well-watered control; AM^+^/WW, mycorrhizal and well-watered; AM^−^/WD, non-mycorrhizal and water deficit; and AM^+^/WD, mycorrhizal and water deficit.

Leaf relative water content (LRWC) ranged 73–80% under WW and 65–69% under WD (Fig. 1), and significantly higher LRWC between treatments ranked as AM^+^/WW > AM^−^/WW > AM^+^/WD > AM^−^/WD (Fig. 1; Table 2).

**Table 2:** Significance of variable variations between *Funneliformis mosseae* and non-*F. mosseae* colonized trifoliate orange (*Poncirus trifoliata*) seedlings under well-watered and water deficit (WD).

Variables	AMF	Water deficit (WD)	AMF×WD
Shoot dry weight	**	**	NS
Root dry weight	**	**	NS
Total plant dry weight	**	**	NS
Leaf relative water content	**	**	NS
Proline concentration in leaves	**	**	NS
Proline concentration in roots	**	**	*
Proline concentration in total plant	**	**	*
Proline content in leaves	**	**	NS
Proline content in roots	**	NS	NS
Proline content in total plant	**	**	NS
P5CS in leaves	**	**	NS
P5CS in roots	**	**	NS
P5CS in total plant	**	**	NS
OAT in leaves	**	**	NS
OAT in roots	*	**	NS
OAT in total plant	**	**	NS
ProDH in leaves	**	**	**
ProDH in roots	*	NS	*
ProDH in total plant	**	**	**

* , *P*<0.05; **, *P*<0.01. Abbreviations: AMF, arbuscular mycorrhizal fungus; NS, not significant; OAT, ornithine-δ-aminotransferase; P5CS, ▵^1^-pyrroline-5-carboxylate synthetase; ProDH, proline dehydrogenase.

**Figure 2:**
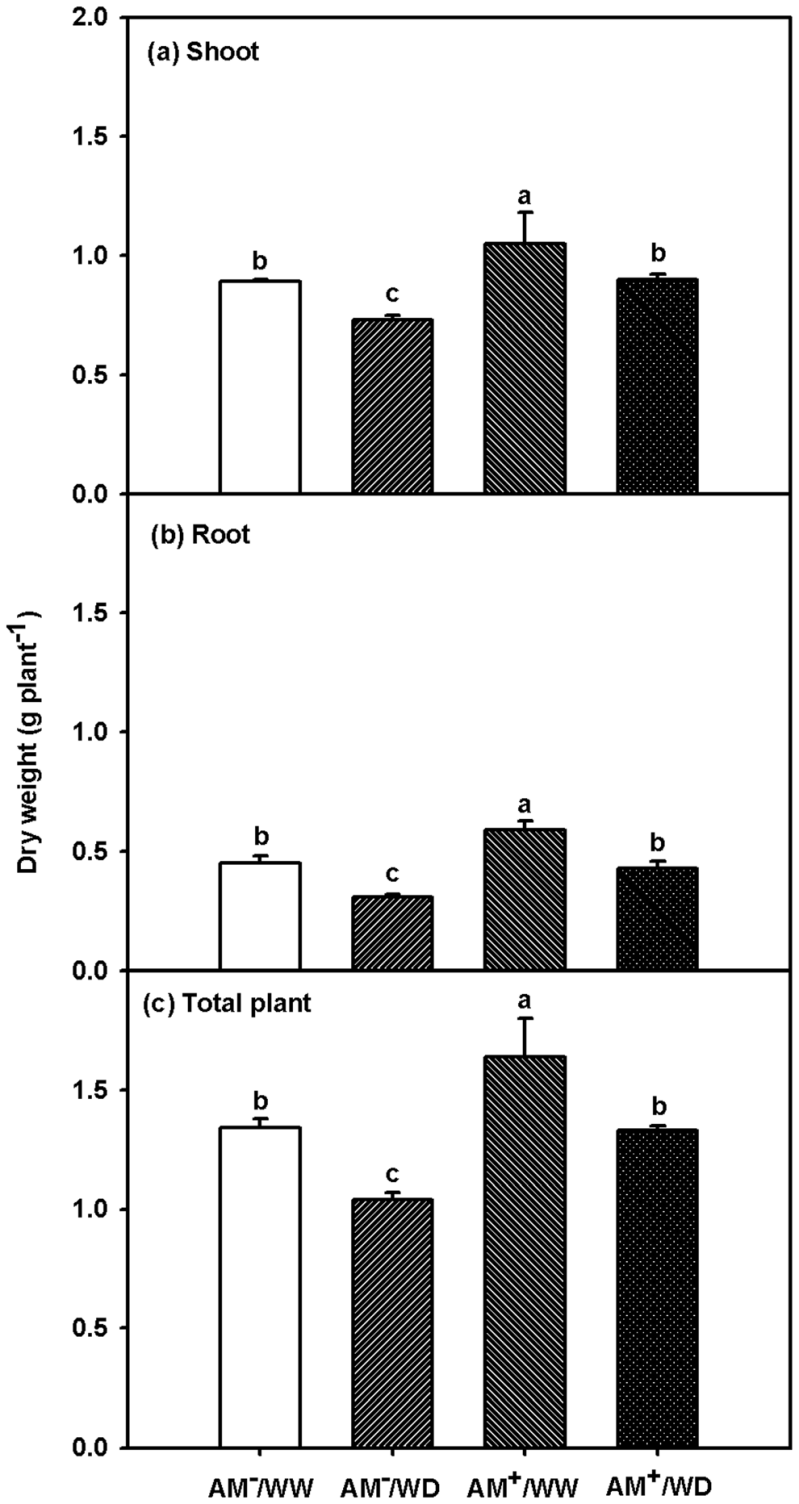
Effects of *Funneliformis mosseae* on shoot (a), root (b), and total plant (c) dry weight of 167-day-old *Poncirus trifoliata* under well-watered and water deficit. Data (means ± SD, *n* = 3) followed by different letters above the bars among treatments indicate significant differences at 5% level. AM^−^/WW, non-mycorrhizal and well-watered control; AM^+^/WW, mycorrhizal and well-watered; AM^−^/WD, non-mycorrhizal and water deficit; and AM^+^/WD, mycorrhizal and water deficit.

Plant total biomass production ranged 1.34–1.64 mg/plant under WW and 1.04–1.33 mg/plant under WD (Fig. 2), and significantly greater biomass production of shoots, roots, and total plant between treatments ranked as AM^+^/WW > AM^+^/WD ≈ AM^−^/WW > AM^−^/WD (Fig. 2a–2c; Table 2).

**Figure 3:**
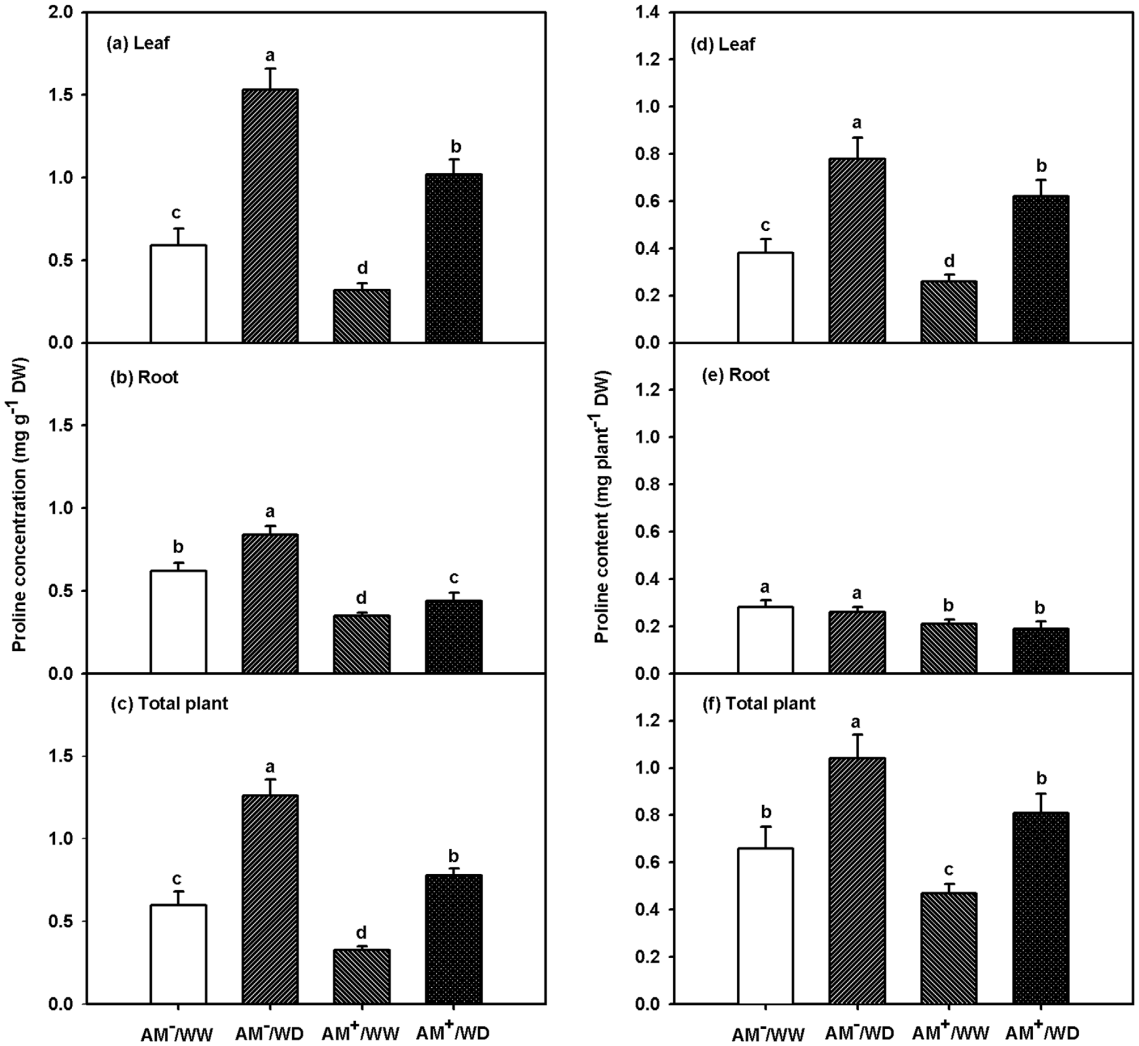
Effects of *Funneliformis mosseae* on proline concentration and content in leaf, root, and total plant of 167-day-old *Poncirus trifoliata* under well-watered and water deficit. Meanwhile, the total plant concentration of proline was calculated as (leaf proline concentration × leaf biomass weight + root proline concentration × root biomass weight)/(leaf biomass weight + root biomass weight). Data (means ± SD, *n* = 3) followed by different letters above the bars among treatments indicate significant differences at 5% level. Abbreviations: AM^−^/WW, non-mycorrhizal and well-watered control; AM^+^/WW, mycorrhizal and well-watered; AM^−^/WD, non-mycorrhizal and water deficit; and AM^+^/WD, mycorrhizal and water deficit.

Proline concentrations ranged 0.32–0.59 mg/g DW in leaves, 0.35–0.62 mg/g DW in roots, and 0.33–0.60 mg g^−1^ DW in the weighted total plant (thereafter ‘total plant’) under WW or 1.02–1.53 mg/g DW in leaves, 0.44–0.84 mg/g DW in roots, and 0.78–1.26 mg/g DW in total plant under WD (Fig. 3a–3c). Significantly higher proline concentrations between treatments ranked as AM^−^/WD > AM^+^/WD > AM^−^/WW > AM^+^/WW in both leaves and total plant (Fig. 3a, 3c; Table 2) whereas as AM^−^/WD > AM^−^/WW > AM^+^/WD > AM^+^/WW in roots (Fig. 3b; Table 2). Meanwhile, proline contents ranged 0.26–0.38 mg/plant DW in leaves, 0.21–0.28 mg/plant DW in roots, and 0.47–0.66 mg/plant DW in total plant under WW or 0.62–0.78 mg/plant DW in leaves, 0.19–0.26 mg/plant DW in roots, and 0.91–1.04 mg/plant DW in total plant under WD (Fig. 3d–3f). Significantly higher proline contents between treatments ranked as AM^−^/WD > AM^+^/WD ≥ AM^−^/WW > AM^+^/WW in leaves and total plant, whereas as AM^−^/WW ≈ AM^−^/WD > AM^+^/WW ≈ AM^+^/WD in roots (Fig. 3d–3f; Table 2).

**Figure 4:**
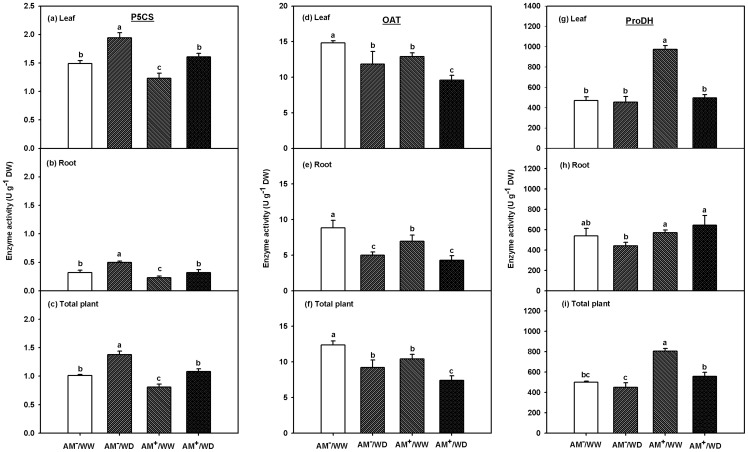
Effects of *Funneliformis mosseae* on activity of ▵^1^-pyrroline-5-carboxylate synthetase (P5CS), ornithine-δ-aminotransferase (OAT), and proline dehydrogenase (ProDH) in leaf, root, and total plant of 167-day-old *Poncirus trifoliata* under well-watered and water deficit. Meanwhile, the total plant activity of proline relevant enzymes was calculated as (leaf activity of proline relevant enzymes × leaf biomass weight + root activity of proline relevant enzymes × root biomass weight)/(leaf biomass weight + root biomass weight). Data (means ± SD, *n* = 3) followed by different letters above the bars among treatments indicate significant differences at 5% level. Abbreviations: AM^−^/WW, non-mycorrhizal and well-watered control; AM^+^/WW, mycorrhizal and well-watered; AM^−^/WD, non-mycorrhizal and water deficit; and AM^+^/WD, mycorrhizal and water deficit.

Activity of P5CS ranged 1.23–1.49, 0.23–0.32 and 0.81–1.01 U/g in leaves, roots and total plant under WW or 1.61–1.94, 0.32–0.50 and 1.08–1.38 U/g in leaves, roots and total plant under WD, respectively (Fig. 4a–4c). Significantly higher activity of P5CS between treatments ranked as AM^−^/WD > AM^+^/WD ≈ AM^−^/WW > AM^+^/WW in leaves, roots and total plant (Fig. 4a–4c; Table 2).

Activity of OAT ranged 12.91–14.82, 6.97–8.85 and 10.41–12.37 U/g in leaves, roots and total plant under WW or 9.57–11.83, 4.27–5.00 and 7.40–9.23 U/g in leaves, roots and total plant under WD, respectively (Fig. 4d–4f). Significantly higher activity of OAT between treatments ranked as AM^−^/WW > AM^+^/WW ≈ AM^−^/WD > AM^+^/WD in leaves and total plant, whereas as AM^−^/WW > AM^+^/WW >AM^−^/WD ≈ AM^+^/WD in roots (Fig. 4g–4i; Table 2).

Activity of ProDH ranged 472–975, 539–572 and 500–805 U/g in leaves, roots and total plant under WW or 455–497, 441–644 and 450–558 U/g in leaves, roots and total plant under WD, respectively (Fig. 4g–i). Significantly higher activity of ProDH between treatments ranked as AM^+^/WW > AM^+^/WD ≈ AM^−^/WW ≈ AM^−^/WD in leaves, whereas as AM^+^/WD ≈ AM^+^/WW > AM^−^/WD ≤ AM^−^/WW in roots and as AM^+^/WW > AM^+^/WD > AM^−^/WD ≤ AM^−^/WW in total plant (Fig. 4g–4i; Table 2).

**Figure 5:**
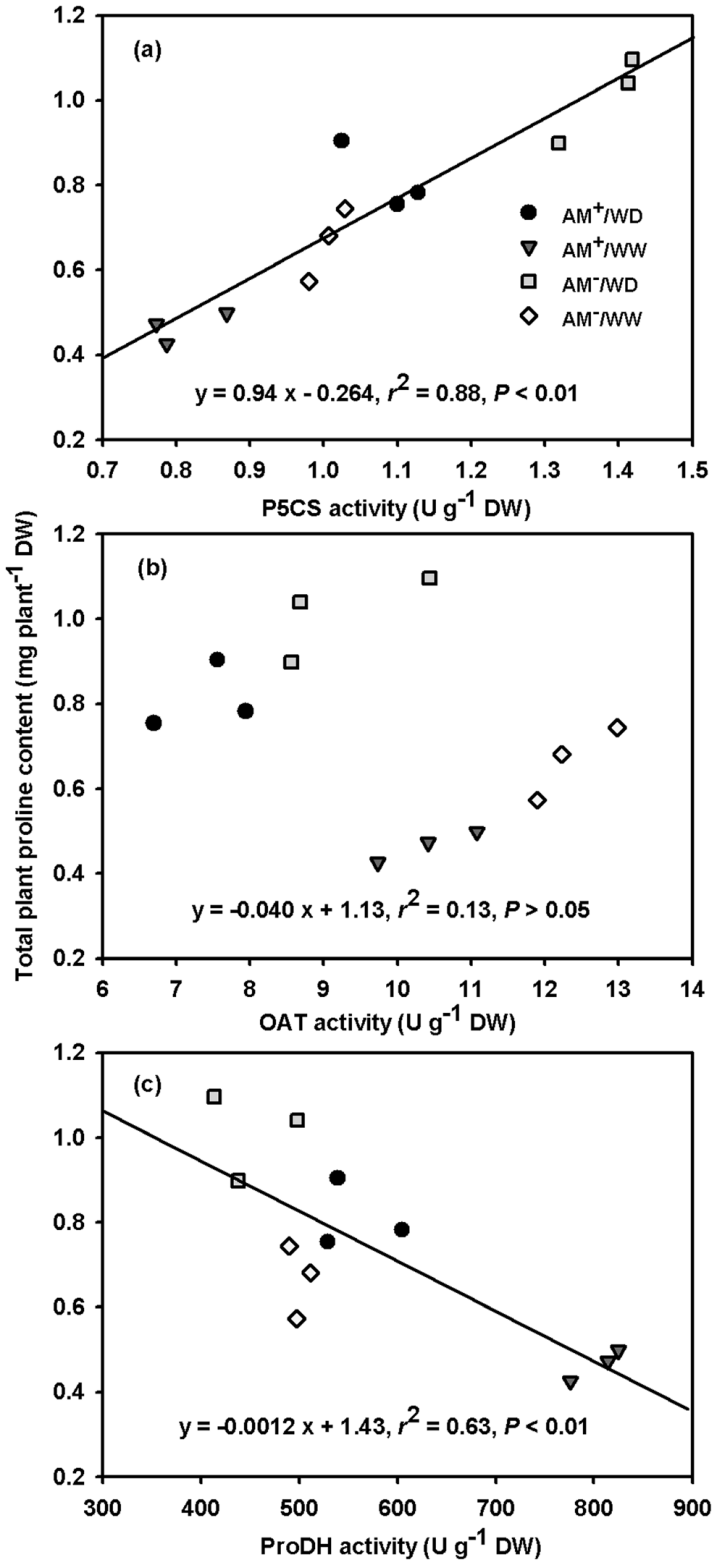
Linear regression between total plant proline content and activity of ▵^1^-pyrroline-5-carboxylate synthetase (P5CS), ornithine-δ-aminotransferase (OAT), and proline dehydrogenase (ProDH) in total plant of *Poncirus trifoliata* under well-watered and water deficit (*n* = 12). Abbreviations: AM^−^/WW, non-mycorrhizal and well-watered control; AM^+^/WW, mycorrhizal and well-watered; AM^−^/WD, non-mycorrhizal and water deficit; and AM^+^/WD, mycorrhizal and water deficit.

The total plant proline content significantly positively correlated with the total plant P5CS activity (*r* ^2^ = 0.88, *P*<0.01) (Fig. 5a), significantly negatively correlated with the total plant ProDH activity (*r* ^2^ = 0.63, *P*<0.01) (Fig. 5c), but did not correlate with the total plant OAT activity (Fig. 5b).

## Discussion

In the present study, WD induced significantly higher total plant (leaf + root) proline accumulation in trifoliate orange seedlings (Fig. 3c, 3f), in company with a general increase of total plant P5CS activity, a decrease of both total plant OAT and ProDH activity (Fig. 4). It suggests that proline accumulation is of great importance in plant responses to WD [3], [4]. In addition, WD caused lower OAT activity of both the AM and no-AM seedlings, suggesting that OAT was not involved in WD-induced proline biosynthesis in trifoliate orange seedlings [31]. Meanwhile, OAT involves in nitrogen transformation from arginine to glutamate-semialdehyde through P5C, which is converted to glutamate by ▵^1^-pyrroline-5-carboxylate dehydrogenase [4]. The net accumulation of proline in AM and no-AM trifoliate orange might involve in the process of plant adaptation to WD. For instance, as a general pattern, in the glutamate and/or ornithine synthetic pathway of proline, WD resulted in a net proline accumulation [3], [32]. Compared to trifoliate orange growing under WW, the present study showed that a greater proline level under WD is dependent on both a higher glutamate synthetic pathway and a lower proline catabolism (Fig. 5).

Under WD proline accumulation was either significantly higher [13]–[16] or lower [9], [17]–[19] in AM than in no-AM plants. In the present study, *F. mosseae* colonization significantly decreased both concentrations and contents of leaf, root, and total plant proline in trifoliate orange seedlings, regardless of water status (Fig. 3). Similar results were found in *Citrus tangerine*, *Erythrina variegata, Knautia arvensis*, and *Trifolium alexandrium* [9], [17], [18], [33]. The present study also showed that under WD, the AM plants presented higher root volume as compared with no-AM control (Table 1), leading to more uptake from soil by roots, and hence meaintaining a higher water status in leaves. As a result, the AM seedlings recorded a higher LRWC than the non-AM seedlings under WD (Fig. 1). Since the drought harm or damage on plant performance was highly related to a water status of AM plants [34], better leaf water status and root volume in the AM plants evidenced that the AM plants were suffered less damage under WD as compared with the no-AM plants. An accumulation of proline has been considered as a sensation of the damage that the plant is suffering [35]. As a result, plants colonized by AMF with a lower proline accumulation would be less harmed under WD and thus more successfully in avoidance of WD [1]. Similar results were found in the *Impatiens balsamina* plants infected by nematodes (*Meloidogyne incognita*) and a mixture of eight AMF species [36]. Therefore, the AM plants might not need to synthetize more proline to tolerate soil water deficit.

In the present study, a significantly lower P5CS and OAT activity of leaf and total plant was found in the AM than in the no-AM trifoliate orange seedlings under both WW and WD (Fig. 4a–4f). The mycorrhizal-mediated lower proline accumulation might result from the inhibition of both glutamate and ornithine synthetic pathways of proline [4]. However, compared with that of the no-AM counterparts, their root P5CS activity of the AM seedlings was significantly higher but the root OAT activity was similar under WD. The root system is the first organ to perceive a stress signal for WD [37]. It suggests that OAT might not be vital to contribute to the proline biosynthesis in AM roots under WD. Total plant proline was significantly positively correlated with total plant P5CS activity, but not total plant OAT activity (Fig. 5a, 5b). As a result, the glutamate synthetic pathway may play a leading role in proline biosynthesis when an AM plant is exposed to WD. Porcel et al. [38] reported that lower *P5CS* transcript expression was in *F*. *mosseae*-colonized soybean and lettuce plants than in no-AMF colonized plants under WD. Proline biosynthesis is usually controlled by *P5CS1* and *P5CS2* gene in plants, and the *P5CS1* can be regulated by abiotic stresses, leading to an enhanced proline biosynthesis in plastids [4]. Further studies are needed to understand the expression of *P5CS1* gene in the proline biosynthesis of AM plants growing under WD.

Another factor contributing to proline accumulation under WD is the catabolism of proline [39], where ProDH catalyses the oxidation of proline to P5C in mitochondria [3]. In the present study, the AMF colonization significantly increased ProDH activity in leaves and total plant of the AM seedlings under WW and in roots and total plant of the AM seedlings under WD (Fig. 4g–4i). The increase of ProDH activity in AM plants may, to some extent, have contributed to a greater proline catabolism under WW or WD, thus leading to a lower proline accumulation. In addition, the *ProDH* gene is rarely expressed and the ProDH protein is more stable than the P5CS protein [40]. However, it is not clear whether a lower proline level in an AM plant under WD is related to the overexpression of *ProDH*.

## Conclusion

In short, inoculation with *F. mosseae* increased the plant growth performance and biomass production but decreased tissue proline accumulation under either WW or WD. The total plant proline significantly positively correlated with plant P5CS activity, negatively with ProDH activity, but not with OAT activity. On the other hand, the AM plants may suffer less from WD due to better leaf water status and root volume, thereby showing lower proline accumulation. In addition, the lower proline accumulation in the AM plants may derive from the integration of the inhibition of glutamate synthetic pathway of proline with an enhancement of proline degradation, no matter whether plants grow under WD or not.
